# Immunogenic Potential of Beer Types Brewed With *Hordeum* and *Triticum* spp. Malt Disclosed by Proteomics

**DOI:** 10.3389/fnut.2020.00098

**Published:** 2020-07-09

**Authors:** Valentina Spada, Luigia Di Stasio, Stefania Picascia, Bernardo Messina, Carmen Gianfrani, Gianfranco Mamone, Gianluca Picariello

**Affiliations:** ^1^Institute of Food Sciences, National Research Council (CNR), Avellino, Italy; ^2^Institute of Biochemistry and Cell Biology, National Research Council (CNR), Naples, Italy; ^3^Research Consortium “Gian Pietro Ballatore,” Palermo, Italy

**Keywords:** beer, barley, wheat, einkorn, allergens, celiac disease, gluten epitopes

## Abstract

The protein/peptide composition of five beer kinds, including two experimental beer-like products brewed with einkorn (*Triticum monococcum*), a beer labeled as “gluten-free,” a traditional all-barley malt and a wheat (*T. aestivum*) containing beer, was characterized with HPLC-ESI MS/MS-based proteomics. To enlarge the characterization of the components, the polypeptides were fractionated according to their molecular size (cut-off 6 kDa). All the beer types contained a variety of polypeptides arising from all the gliadin subfamilies (α-/β-, γ-, and ω-gliadins) able to induce an immune response in celiac disease (CD) patients in addition to a panel of IgE-reactive food allergens. Wheat storage proteins were heavily hydrolyzed in the beer samples brewed with einkorn. The presence of gluten-like fragments, also including the 25-mer and 33-mer-like of α-gliadin, was confirmed in beer brewed with barley and wheat malt as well as in the gluten-free beer. Both CD-toxic and allergenic peptides of all beer samples were drastically degraded when subjected to a simulated gastroduodenal (GD) digestion. After *in vitro* digestion, the level of gluten-like peptides assayed with the G12 competitive ELISA, was below the threshold (20 ppm) for a food to be considered as “gluten-free.” A few gliadin-derived epitopes occurred in the digests of beers crafted with wheat or Norberto-ID331 line of einkorn. In contrast, digests of all barley malt and gluten-free beers did not contain detectable gluten-like epitopes, but only minor fragments of hordeins and IgE-reactive food allergens. All beer samples evoked a weak immune response on gliadin-reactive celiac T cells isolated from intestinal biopsies of celiac patients. Compared to undigested polypeptides, the response was markedly reduced by GD digestion. Although the consumption of a moderate amount of beer brewed with barley or einkorn could deliver a relatively low amount of CD-toxic epitopes, the findings of this study emphasize the urgent need of a reliable and accurate quantification of gluten epitopes in all types of beer, also including the gluten-free one, to compute realistically the contribution of beer to the overall gluten intake, which can be responsible of intestinal tissue damages in celiacs.

## Introduction

Protein content of beer ranges from 0.2 to 2.9 g/L depending on the brewing style (1). Despite its relatively low content, the proteinaceous fraction has a primary impact on the sensory traits, affecting foam firmness, haze formation, texture, colloidal stability and color. Beer ranks first among the alcoholic beverages consumed worldwide. Beyond the economic interest, beer is an interesting model of deeply processed cereal-based matrix and, furthermore, it can contain immunologically active polypeptides responsible of IgE-mediated allergic reactions or gluten-related diseases, such as celiac disease (CD). These aspects have motivated a detailed characterization of beer proteins since decades.

The large majority of beer proteins originates from malt barley (*Hordeum vulgare*) or from wheat (*Triticum aestivum*) in the case of wheat beer (or Weissbier), which is popular in Belgium and Germany (2). Proteins derived from other possible malted or unmalted cereal adjuncts and from yeast occur at a much lesser extent (3).

Partially digested storage proteins of *Triticeae* (wheat, barley, and rye), especially the 60–70% ethanol soluble fraction of wheat (gliadins), constitute the trigger factor of CD, which is an autoimmune-mediated enteropathy of the small intestine. In a classic study, Ellis et al. (4) detected relatively high amounts of hordeins (i.e., storage proteins of barley) containing gliadin-like epitopes in both malt and beer, which entail the precautionary exclusion of beer from the diet of celiacs. Subsequent analyses based on two-dimensional electrophoresis-mass spectrometry (MS) proteomics excluded the presence of major amounts of intact prolamins in beer (5–7). On the other hand, data obtained with high performance liquid chromatography-tandem mass spectrometry (HPLC-MS/MS) gel-free proteomics and peptidomics demonstrated that beer contains significant amounts of polypeptides toxic for celiac subjects, especially plentiful in the low molecular weight fraction (3, 8, 9), which should support preclusion of beer for celiacs. More advanced proteomic strategies based on HPLC-MS/MS with data dependent or independent acquisition have dramatically enlarged the beer protein inventory, up to thousands of gene products (10, 11). However, these investigations substantially confirm that potentially harmful components of beer almost exclusively are soluble hordein fragments released by proteolytic events. The most abundant unhydrolyzed proteins in beer are cereal serpins, lipid transfer proteins and α-amylase/trypsin inhibitors, all of which being potentially responsible of adverse reactions with mechanisms distinct from those triggered by prolamins-derived CD-toxic peptides (12).

The reliable quantification of gluten-like epitopes in beer is still debated. In general, quantification of gluten in hydrolyzed/fermented foods or beverages is hindered by a series of factors, especially related to the lack of appropriate reference standards (13). Multiplicity of barley varieties utilized for brewing, heterogeneity of the prolamins, variable degree of proteolysis, matrix interferences, ample dynamic range of proteolytic fragments, process induced modifications (e.g., non-enzymatic glycation) are additional issues complicating the accurate estimation of beer gluten content (14). Due to these drawbacks, it is clearly emerging that immunochemical methods such as both sandwich and competitive R5 ELISA, which are the most exploited and the unique recommended by food control authorities, could be unreliable for quantifying gluten in beer, because results are often affected by discrepancies spanning over several orders of magnitude (15). In turn, the complexity of more than 1,200 known gluten epitopes (16, 17) and many others not cataloged yet, severely challenges the potentiality of MS-based methods to assess the suitability of foods and beverages for celiacs (10). Thus, uncertainty remains about the actual amount of immunotoxic peptides and about health risk associated with moderate beer consumption by CD subjects.

Several kinds of beers specifically produced for celiacs are brewed with non-gluten-containing cereals (e.g., sorghum, millet, rice, corn) or pseudocereals (e.g., buckwheat). In general, these beers find low acceptance by consumers as they lack the distinctive sensory traits imparted by malted barley.

In order to elicit an inflammatory response in celiacs, gluten peptides with a minimum length of 9 amino acid residues need to survive human gastrointestinal digestion and to reach the intestinal lamina propria where they come in touch with HLA-DQ2/8 immunocompetent cells (18). Thus, hydrolytic enzymes able to split gluten peptides in fragments smaller than 9 amino acids would in principle abolish the CD immunoreactivity. To this purpose, prolyl endopeptidases (PEP) from several organisms, especially from *Aspergillus niger* (AN), are marketed as dietary supplements intended to destroy possible gluten contaminants eaten with the gluten-free foods (19). AN-PEP is currently used to brew commercial beer from barley malt, which according to the European Commission Regulation No. 41/2009 and Food and Drug Administration Final Rule 47154/2013 that transpose the Codex Alimentarius indications, can be labeled as “gluten-free” if gluten <20 ppm [normative indications can be found in reference no. (20)]. However, the opportunity that consumers could be informed and distinguish between food that is made exclusively from ingredients naturally free of gluten and other prepared and/or processed in order to reduce overall gluten is debated. This issue is extremely relevant, due to the current limitations for the accurate quantification of gluten in processed samples. Thus, in USA, beers intended for celiacs are categorized as gluten-free or gluten-reduced beers.

In the attempt of recovering the taste of ancient beer and to diversify the beer kinds on the market, several small breweries have started to brew beer with einkorn (*Triticum monococcum*) malt, alone or combined with barley malt. Proteins from some lines of einkorn, which lacks the genome B and D of hexaploid wheat, have a reduced T cell stimulatory potential and do not induce activation of the innate immune response in CD subjects. For this reason, einkorn has been suggested as an alternative cereal for individual genetically at risk of CD (21). Thus, although einkorn-based products are not adequate for CD subjects, einkorn beer might have reduced impact in terms of immunotoxic sequences, also because its gluten appears to be more easily degraded by gastrointestinal proteases (21, 22).

In this explorative study, we characterized by HPLC-MS/MS the high-(HMW) and low-(LMW) molecular weight (poly)peptide fractions of five model commercial beers, including German Weissbier, typical all-barley malt, barley-einkorn blend, all-einkorn malt and AN-PEP-brewed gluten-free barley-based types. Special focus was on CD-toxic proteins as well as on potential IgE food allergens occurring in the five beer types. Beer samples were subjected to an *in vitro* simulated digestion using a static *in vitro* gastro-duodenal model, to evaluate the bioaccessibility of possible CD-toxic and allergenic (poly)peptides.

## Materials and Methods

### Samples and Chemicals

Three commercial trademarks of all-barley malt (Peroni Riserva), Weissbier (Franziskaner) and a barley malt-based gluten-free beer (Tennent's gluten-free) were purchased in a local supermarket. Based on the producers' technical information, Weissbier is brewed according to the German style, using 50–60% of malted wheat as a grist adjunct. The gluten-free beer is produced with barley malt and using AN-PEP to hydrolyze gluten-like peptides. According to the USA labeling system, this beer should be rather labeled as “gluten-reduced” beer. Non-commercial experimental fermented and non-filtered beer-like products, namely “Rossa di Hammurabi,” brewed exclusively with *T. monococcum* malt, Hammurabi line, and “Birra di Monococco” brewed with 50% barley malt and 50% *T. monococcum* malt, Norberto-ID331 line, were experimentally produced by the Research Consortium “G. P. Ballatore” in a Sicilian Agrofood farm located in Salemi (Trapani), Italy. Thereinafter these beer-like products will be indicated simply as “Hammurabi” and ID331 beers. Farm assured that Hammurabi einkorn beer was brewed exclusively with malt of *T. monococcum* Hammurabi. For brewing with *T. monococcum*, malted cereals were grossly milled. Crushed kernels were steeped in water (approximate proportion: 1 kg malt for 5 L water) and temperature was varied according to the following steps: 55°C for 10 min (1st step, protein rest); 62°C for 60 min (2nd step, α-amylase rest); 72°C for 10 min (3rd step, β-amylase); 78°C for 10 min (4th step, mash out); 105°C boiling for 60 min (5th step, hopping at this stage); whirlpooling (6th step, sedimentation); 16°C for 8 days (7th step, fermentation); cooling at 2°C (8th step, sedimentation); bottling and addiction of rectified concentrated wort (9th step, re-fermentation). Chemicals and HPLC-MS-grade solvents were purchased from Sigma (St. Louis, MI, USA).

### Purification of Beer Protein and Peptide Fractions

Protein and peptides were purified from degassed beer samples as previously described (2). In order to retain possible undissolved particles, proteins were precipitated overnight at 4°C with trichloroacetic acid up to final 20% (w/v). The pellet obtained after centrifugation (5000 × g) was re-dissolved in 1 mL denaturing/reducing buffer (6 M guanidine, 50 mM Tris, 10 mM dithiothreitol, DTT, pH 8.0) and incubated for 30 min at 55°C. Proteins were Cys-alkylated with 55 mM iodoacetamide at room temperature in the dark. The HMW (>6 kDa) protein fraction of each beer type was purified using G-25 Econo-Pak 10 DG columns (Biorad, Milan, Italy; exclusion limit 6 kDa), eluting with 50 mM ammonium bicarbonate, pH 7.8. After quantification with the modified micro-Lowry assay (kit from Sigma-Aldrich), aliquots of the beer protein fraction (>6 kDa) were digested overnight at 37°C with proteomic-grade modified trypsin (Sigma) at a 1:100 (w/w) enzyme–substrate ratio. The low molecular weight (<6 kDa) peptide fractions were separated with Econo-Pak 10 DG columns from different aliquots of untreated beer, eluting with 1% acetic acid. Peptides were quantified with the micro-Lowry assay and lyophilized. An aliquot of the peptide fraction (<6 kDa) of each beer was digested in the same conditions above with proteomic-grade chymotrypsin (enzyme from Sigma), in order to enlarge the coverage of the peptide sequences. In fact, chymotrypsin is more effective than trypsin to hydrolyze prolamins, due to the shortage of lysine and arginine residues (23). Prior to analysis, tryptic (or chymotryptic) peptides, as well as the LMW peptide fractions (<6 kDa), were concentrated and purified from polar compounds using C18 Sep-pak cartridges (Waters, Milford, MA, USA) eluting with 70% acetonitrile/ 0.1% TFA and finally vacuum-dried.

### Simulated Gastroduodenal Digestion of Beer

Beer samples were subjected to a simulated gastroduodenal (GD) static *in vitro* digestion, skipping the oral phase. Simulated gastric fluid (SGF) and simulated intestinal fluid (SIF) were prepared according to the Infogest harmonized conditions (24, 25). Digestion steps were carried out in a shaking incubator at 37°C and 170 rpm. Briefly, for the gastric phase 2 mL of beer samples was diluted with 1.6 mL of SGF 1.25x. Liposomes, freshly prepared with lecithin (Sigma), were added up to 0.17 mM in the final solution. The pH was adjusted to 2.7, porcine pepsin was included at 2000 units/mL final concentration and the solution, adjusted to 4 mL with water, was incubated for 2 h at 37°C. Pepsin hydrolysis was stopped by raising the pH to 7.0 with 1 M sodium bicarbonate. Simulated duodenal digestion was carried out for 2 h at 37°C after duplicating the volume with SIF, containing bile salts (10 mM final on the basis of cholic acid) and pancreatin at 100 U/mL based on the trypsin activity [7 U/mg of pancreatin as N-4-tosyl-L-arginine methyl ester (TAME) activity]. Immediately after the duodenal phase, peptide digests were purified with C18 reverse-phase prepacked cartridges (Sep-Pak, Waters, Milford, MA, U.S.A.), washing extensively with 0.1% (v/v) trifluoroacetic acid (TFA) and eluting with 70% acetonitrile/0.1% TFA. Peptides were vacuum-dried and finally lyophilized.

### G12 Assay

Gluten content of beer samples before and after simulated GD digestion was determined with the competitive G12 assay kit (Biomedal SL, Sevilla, Spain), as previously described (2). Standard gliadin at various dilutions (0–120 ng/mL) was used to build a calibration curve. Before analysis, duodenal proteases were inhibited with 1 mM final Pefabloc® (Sigma-Aldrich) and samples were warmed for 40 min at 50°C, to minimize possible protein–polyphenols interactions. Afterward, beer aliquots were diluted (1:50–1:200) in the dilution buffer (room temperature) and assayed in triplicate. At the dilution considered the upper limit for gluten quantification was 80 ppm. Statistical analyses were performed with the Excel 2013 software (Microsoft Co., WA, USA).

### Celiac T Cell Functional Assay

T cell lines (TCLs) highly reactive to wheat gliadin proteins were generated from jejunal biopsies of *N* = 3 HLA-DQ2.5 celiac patients, as previously reported (26, 27). Briefly, mucosal cells were *in vitro* stimulated with an enzymatic (pepsin-chymotrypsin) digest of gliadin proteins (PC-gliadin) in complete medium (X-Vivo 15 medium supplemented with 5% AB-pooled human serum and antibiotics, all purchased by Lonza, Canberra Australia). PC-gliadin digest used for the establishment of TCLs were deamidated by recombinant tissue transglutaminase-2 (tTG) enzyme (Sigma), as detailed below. To assess the antigen specificity, T cells (3 × 10^4^) were co-incubated in complete medium with allogeneic HLA-DQ2.5 immortalized B-cells (B-LCL) used as antigen presenting cells (1 × 10^5^), with each beer sample or PC-gliadin, used as internal positive control in 96-well round bottom plates (200 μL/well). Peptides were purified from beer before and after simulated GD digestion using C18 Sep-pak cartridges as described above and deamidated with recombinant tTG (Sigma) at a 1/10 enzyme/substrate ratio, 4 h at 37°C in 50 mM Tris–HCl buffer (pH 6.8) containing 5 mM CaCl_2_, 10 mM NaCl and 10 mM DTT. The reaction was arrested by incorporating EDTA up to final 10 mM and deamidated peptides were assayed at 100 μg/mL concentration. As read-out of T cell immunogenicity, IFN-γ production was measured in triplicates on cell supernatants after 48 h of cell incubation by a classic sandwich ELISA. Purified and biotin-conjugated anti-IFN-γ MoAbs were purchased from Mabtech (Nacka Strand, Sweden).

### Nanoflow-HPLC-ESI-MS/MS

Nanoflow-HPLC-MS/MS analyses were performed using an Ultimate 3000 nanoflow ultra-HPLC (Dionex/Thermo Scientific, San Jose, CA, USA) coupled to a Q-Exactive Orbitrap mass spectrometer (Thermo Scientific) through a nano-electrospray ionization source. Tryptic peptides generated from the protein fractions or free peptides isolated from beer samples were reconstituted in 0.1% formic acid and nearly 2 μg were loaded onto the column through Acclaim PepMap 100 trap columns (75-μm i.d. x 2 cm; Thermo Scientific) using a FAMOS autosampler (Thermo Scientific). Peptides were separated using an EASY-Spray™ PepMap C18 column (2 μm, 25 cm × 75 μm) with 2-μm particles and 100-Å pore size (Thermo Scientific), applying a 2–50% gradient of B over 120 min after 10 min of isocratic elution at 2% B and a constant flow rate of 300 nL/min. Eluent A was 0.1% formic acid (v/v) in LC-MS-grade water, and eluent B was 0.1% formic acid (v/v) in AcN. MS1 precursor spectra were acquired in the positive ionization mode scanning the 1800-300 *m/z* range with resolving power of 70.000 full width at half maximum (FWHM), an automatic gain control (AGC) target of 1 × 10^6^ ions, and maximum ion injection time of 256 ms. The spectrometer operated in full scan MS1 and data-dependent acquisition mode, selecting up to the 10 most intense ions for MS/MS fragmentation and applying a 12 s dynamic exclusion. Fragmentation spectra were obtained at a resolving power of 17.500 FWHM. Ions with one charge or more than six were excluded from the MS/MS selection. Spectra were elaborated using the software Xcalibur version 3.1 (Thermo Scientific).

### Bioinformatics

LC-MS/MS raw data were analyzed with the Proteome Discoverer software version 2.1 (Thermo Scientific), purchased with the spectrometer. The same HPLC-MS/MS runs were converted into mgf siles using the ProteoWizard 3.0 software (http://proteowizard.sourceforge.net) and analyzed with the Batch-Tag Web tool of Protein Prospector (https://prospector.ucsf.edu). The searches were taxonomically restricted to *Hordeum, Oryza, Saccharomyces, Triticum*, and *Zea* protein databases downloaded from UniprotKB (updated in November 2019). For the analysis of tryptic peptides Cys-carbamidomethylation was included as a constant modification and the possibility of semitryptic cleavages was allowed. Two trypsin missed cleavages were allowed. For the identification of free peptides, the search conditions included unspecific cleavage and no static modification. In both cases, methionine oxidation and pyroglutamic acid at N-terminus glutamine were selected as variable modifications.

In all cases, the mass tolerance value was 10 ppm for the precursor ion and 12 ppm for MS/MS fragments. Peptide Spectrum Matches (PSMs) were filtered using the target decoy database approach with an e value of 0.01 peptide-level false discovery rate (FDR), corresponding to a 99% confidence score.

Peptidomic data were visualized with the open source Peptigram web application (http://bioware.ucd.ie/peptigram).

### Statistics

Statistical significance was assessed with a two-tailed paired Student's *t*-test (*p* < 0.05). Analysis were carried out using both GraphPad (GraphPad Software Co., San Diego, CA) and Microsoft Excel 2016.

## Results and Discussion

The protein/peptide composition of five beer types was comparatively characterized using high resolution and high sensitivity nanoflow-HPLC-ESI MS/MS. Compared to previous analyses, the improved analytical performance was expected to enlarge the inventory of the protein species identified in all-barley malt beer and Weissbier (2, 8).

Using more or less sophisticate pre-fractioning strategies coupled to proteomic analysis it has been demonstrated that beer contains up to hundreds or even thousands minor gene products (4, 8, 9). However, in this work we were interested to identify those species occurring at relatively significant amount, which in principle could contribute to the CD-toxicity and immunogenic potential of beer. For this reason, apart from the size-fractionation in high- and low-molecular weight polypeptides (exclusion limit 6 kDa), beer polypeptides were not further separated prior to analysis. The possible presence of gluten-like proteins/peptides was also monitored in a commercial beer labeled as “gluten-free,” which is brewed with barley malt and probably with minor adjuncts of other cereals followed by hydrolysis with AN-PEP. The characterization of proteins in beer brewed with pure or combined einkorn is new, to the best of our knowledge.

### High-Molecular Weight Protein Fraction

Malting, which is a controlled germination of the caryopses, activate at least forty proteases that concur to hydrolyze storage proteins, also releasing starch granules. Proteolysis “selects” the relatively resistant metabolic proteins, while susceptible proteins are extensively degraded.

Cereal prolamins are scarcely or not soluble in the low-alcohol solution of beer. Thus, intact prolamins are for the most removed by precipitation and filtration steps. In contrast, large soluble proteolytic domains of prolamins can be released into beer during mashing. Based on the size-fractioning and sample preparation workflow of this study, it is not possible to distinguish possible traces of intact prolamins from their large proteolytic fragments, which however in general retain the immunogenic potential.

The proteins identified at high confidence in the HMW fraction of the five beer types are cataloged in Table S1. More than 400 gene products were identified in Weissbier, 254 of them detected with at least 2 matching peptides. The top-scoring protein was an α/β-gliadin identified with 97 unique peptide spectrum matches (PSMs), which mapped almost the entire protein sequence. All the subfamilies of wheat prolamins (both gliadins and glutenins) were represented in Weissbier with multiple PSMs. Serpin, non-specific-lipid transfer protein (LTP) and α-amylase/trypsin inhibitor (ATI) isoforms from both wheat and barley were among the most represented proteins, as previously reviewed (12). Most of the gene products identified in all-barley malt beer where from *Hordeum* spp., as expected. A few entries belonging to different cereal species most likely were the result of the identification by homology, due to the incompleteness of the specific protein databases. In all-barley malt beer, barley Z4-serpin and LTPs were among the main proteins, both being established food allergens capable of inducing IgE-mediated adverse reactions (28). D- and γ-hordeins, which are relatively minor classes of barley prolamins, were retrieved with 70 and 50 PSMs, respectively. Notably, D-hordeins share structural homology with wheat HMW glutenins and are immunoreactive to the serum IgA of CD individuals (8). B-type hordeins occurred at intermediate levels, based on the number of PSMs, whereas only a few fragments of C-hordeins were detected, due to their substantial insolubility in the water-based solution of beer (9). The great majority of 246 proteins identified in the HMW fraction of Hammurabi (brewed with einkorn malt) was specific to *T. monococcum*. Many of these entries were assigned to ordinary wheat (*T. aestivum*) by homology, due to the incomplete annotation of einkorn gene products. However, the most abundant proteins of barley malt beer, namely Z-4 serpin, LTP and ATI, were confidently identified with multiple PSMs in Hammurabi beer, suggesting that barley malt could be an accidental contaminant or it had been deliberately used at limited amount for brewing. As expected, ID331 beer, which is brewed with a blend of malted barley and einkorn, contained proteins from both species. Gliadins and glutenins (or hordeins in the case of ID331) belonging to multiple respective subfamilies were among the top-scoring protein entries of both einkorn-containing beer types. Notably, avenin-like proteins (ALPs) were among the most represented proteins either in einkorn-containing samples or in the other beer types. Although described in beer since relatively recent times (29), ALP isoforms could be numbered among the main proteins of beer, also occurring as unhydrolyzed polypeptides at variable amount depending on the beer type (3, 9). By a structural standpoint, ALPs contain an α-amylase inhibitor-like domain and most likely are functionally related to ATI, as they all behave as beer foam-stabilizing proteins (30). An additional group of proteins nearly neglected in previous proteomic investigations, but found ubiquitous in the beer samples analyzed herein, was constituted by dehydrins. These proteins belong to the late embryogenesis abundant proteins, a numerous group of versatile hydrophilic cereal proteins, involved in the protection from abiotic stress such as desiccation or temperature injury (31).

In line with previous findings (32), the gluten-free beer contained D- and γ-type hordeins, identified with multiple PSMs, as well as B3- and B-hordeins identified with only 2 or 1 PSMs, respectively. The origin of gluten-free beer, which is brewed essentially with barley malt, clearly emerged from the pattern of proteins identified, as they were the typical polypeptides of barley malt beer (e.g., Z4-barley serpin, LTP and ATIs). Overall, 111 gene products were identified in the HMW fraction of gluten-free beer, 64 of which identified with at least 2 PSMs. However, gluten-free beer also contained peptides arising from wheat gluten proteins, likely due to an undeclared wheat adjunct rather than being the result of identification by homology, because the PSMs matched with high confidence *Triticum*-specific sequences including gliadin, glutenin, and metabolic proteins.

### Low-Molecular Weight Protein Analysis

More than 80% of the polypeptide fraction of beer consists of variously sized products of protein hydrolysis (3, 33). Many of these peptides with more than 9–10 amino acid residues could retain the CD-toxic or immunogenic potential. Direct identification of these peptides usually is a challenging task, due to large distribution of their molecular weight, the non-tryptic nature, possible events of non-enzymatic glycation, and incompleteness of the databases available. In addition, very large (>40 residues) peptides are hardly identified due to poor fragmentation. A variable number of peptides was identified in the different beer types, ranging from a few (17 peptides in einkorn ID331 beer) to several hundred units (645 in Weissbier). The multiplicity of peptides released is clearly related to both nature of raw material and brewing technology. The list of proteins identified by analyzing free peptides in beer samples is reported in Table S2. Interestingly, 295 peptides belonging to 71 gene products were identified in gluten-free beer, among which 37 peptides of an α-gliadin, besides a variable number of fragments from other gliadin subfamilies and from glutenin subunits. Hordein peptides, especially arising from D-hordeins, were the most numerous in all-barley malt conventional beer (8). Peptides in beer produced with combined barley and ID331 einkorn line matched practically only proteins from the *Hordeum* genus, suggesting that during brewing most of the *T. monococcum*-deriving proteins could be extensively hydrolyzed into small peptides, which remained unidentified. The predominant presence of hordeum-specific derived fragments in the Hammurabi beer, brewed with *T. monococcum* and probabe barley contaminations, even more strikingly supported the concept that einkorn storage proteins could be very susceptible to proteolysis (21).

**Figure 1:**
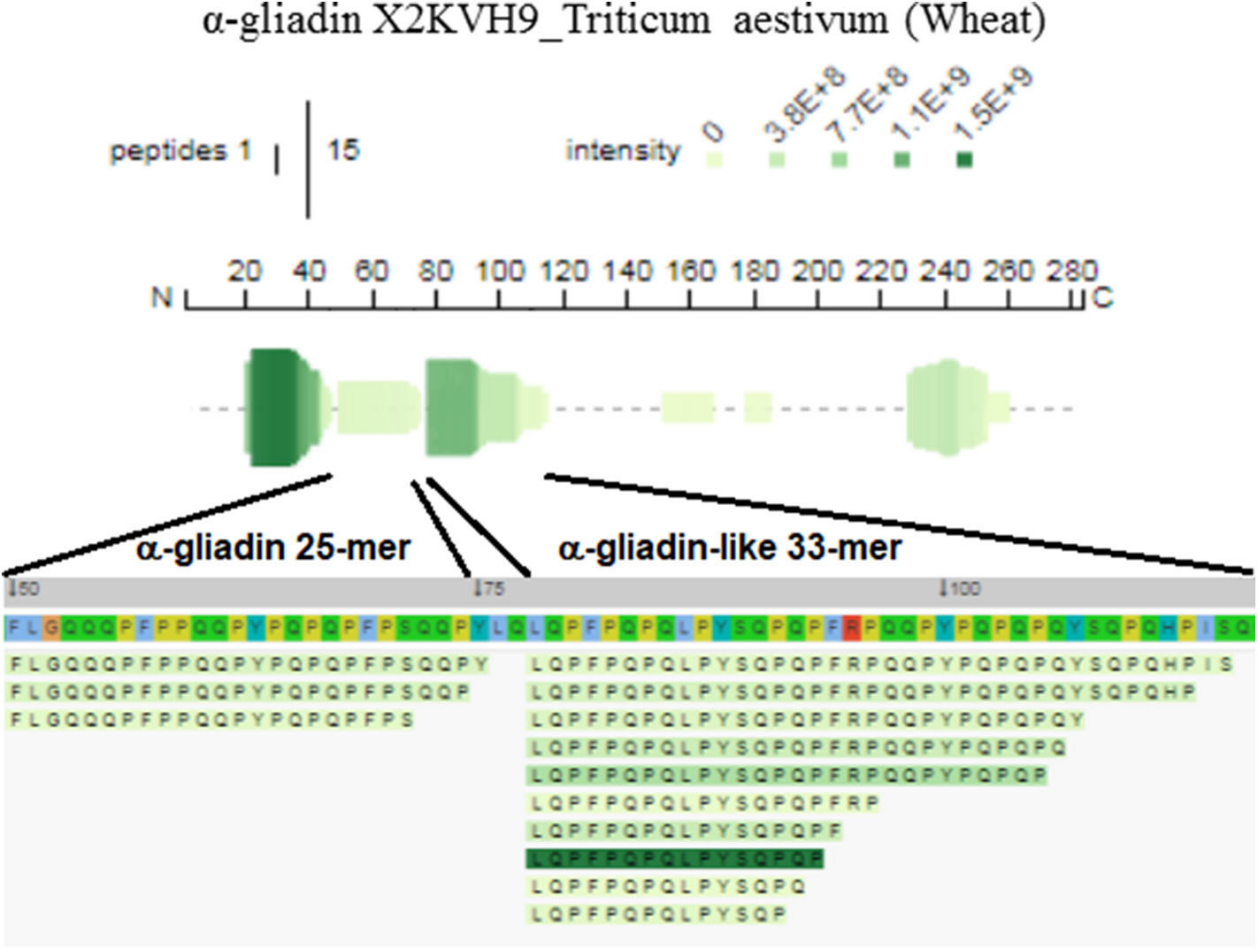
Profile and peptide alignment maps relevant to the epitopic 25- and 33-mer-like domains of α-gliadin detected in the gluten-free beer, visualized with the Peptigram tool. Position in the polypeptide chain includes the signal peptide (1–20 residues).

While the presence of gluten-derived sequences in barley and wheat-containing beer was somehow expected (2), the presence of gluten epitopes in gluten-free beer deserves a closer inspection. The LMW fraction of gluten-free beer contained the QLQPFPQPQLPYSQPQP peptide deriving from an α-gliadin domain. This peptide overlaps a fragment of the α2-gliadin 33-mer, except for the P → S and C-terminal L → P substitutions. The 31-43, 31-49, and 31-55 toxic sequences of α-gliadin also occurred in gluten-free beer. The sequences of the α-gliadin identified originated from the *T. aestivum* genome, although gliadins and glutenins from *T. monococcum* and *T. urartu* were retrieved as well. Figure 1 displays the epitopic regions of α-gliadin detected in the LMW fraction of gluten-free beer.

Sollid et al. (16) classified the T cell epitopes eliciting CD according to nine amino acid-residues core regions, which are shared from most of the DQ2 and DQ8 restricted epitopes.

At least three of the identified peptides of γ-gliadin in gluten-free beer harbored the nine-residues core epitope DQ2.5-glia-γ2 (IQPQQPAQL, the underlined Q being substrate for tissue transglutaminase 2-deamidation). One of the peptides (QPQQPFPQPQQPQ) contains a motif structurally correlated to DQ2.5-glia-γ5 (QQPFPQQPQ). Eight amino acids-motifs (QQPFPQQP) included this latter core epitope in several matching B-hordein-derived sequences. Numerous fragments of LMW glutenin-subunits encrypted the DQ2.5-glut-L2 core epitope and sequences related to the DQ2.5-glut-L1. The C-term domain of the C-hordein QPQQPFPQPQQP fragment overlapped for the first eight residues the DQ2.5-hor-1 (formerly referred to as hor-α9) core epitope, with sequence PFPQPQQPF.

To extend the inventory of the beer peptides, the LMW fractions (< 6 kDa) of all beer samples were re-analyzed after digestion with chymotrypsin, in order to split very large polypeptides not identified by MS/MS. Table S3 reports the list of proteins identified through HPLC-MS/MS analysis of chymotryptic digests in all beer samples. As concerns gluten-free beer, notably, no peptide from the region of immunodominant α-gliadin 33-mer had been detected in the analysis of HMW polypeptides. In contrast, free peptides and chymotryptic digests contained several peptides encrypting the core epitopes of the α-gliadin 33-mer, such as DQ2.5-glia-α1a PFPQPQLPY and PQPQLPYSQ, this latter being highly homolog to DQ2.5-glia-α2 (PQPQLPYPQ). The PQPQPF sequence corresponding to the C-terminal hexamer of α-gliadin 33-mer, which belongs to a CD-toxic domain as demonstrated by *in vivo* studies (34), also occurred in numerous peptides. All the five-residues epitopes recognized by the Mendez R5 antibody were found in numerous peptides.

Thus, in agreement with recent findings (32), the beer labeled as gluten-free, which is crafted with an enzymatic treatment aimed at removing gluten, can contain a multitude of variously sized polypeptides encrypting the immunotoxic epitopes that trigger CD as well as highly homologous sequences. Strikingly, the 13 residues-long N-terminal fragment of α2-gliadin 33-mer has been detected in beer brewed with even high dose of PEP, along with immunotoxic hydrolyzed peptides of HMW glutenins (13). These latter authors also emphasized the possibility that additional immunotoxic peptides remained undetected due to several analytical drawbacks. The concerns related to the consumption of beer crafted with PEP from barley or wheat malt have been recently reviewed (14).

In general, the analysis of the LMW fractions following chymotryptic digestion confirmed the occurrence of hordeins (D- and γ3-subfamilies) in all-barley malt beer (8). The identification of HMW glutenin subunits in beer based on cereals other than wheat was most likely justified by the homology with D-hordeins. Four peptides deriving from the 16-47 domain of barley LTP1 were also identified in the all-barley malt beer. The chymotrypsinized LMW fraction of Weissbier contained the highest number of identified peptides, overall deriving from 121 gene products of wheat, barley and, only for a small fraction, yeast. Interestingly, also in this case the top-scoring protein entry was an α/β-gliadin, which was identified with 58 peptides, including a 31 amino acid residues-long fragment overlapping the domain of the immunodominant 33-mer peptide. Fragments from gliadins and hordeins belonging to all the respective subfamilies as well as LTP1 and Z-serpin isoforms from both wheat and barley were detected. The analysis of chymotryptic digests confirmed that, like the conventional all-barley malt beer, the LMW fraction of einkorn-based beers was dominated by D-hordein (33). Confirming data obtained from the analysis of the HMW fraction, einkorn beer samples as well as all-barley malt and Weissbier contained several peptides from barley dehydrin DHN4.

### Potential Immunogenic and Allergenic Proteins in Beer Samples

The most abundant proteins of beer have structural traits that confer them thermal and proteolytic stability. In principle, it is possible that these proteins or their large immunologically active fragments reach the small intestine, where they can come in touch with immunocompetent cells. In Table 1 are listed the established or putative food allergens and CD-toxic gluten proteins detected in beer samples as unhydrolyzed gene products or identified through their proteolytic fragments. Serpins and ATIs are serine protease inhibitors; LTPs are presumed cysteine proteases inhibitors (35). By virtue of their proteolytic stability, these proteins accumulate during brewing, as the result of the degradation of other cereal proteins. LTPs, almost ubiquity amongst plants, are considered a class of “pan-allergens” and are well-established determinants of even severe and life-threatening IgE-mediated allergies. Several other beer proteins can be potent triggers of food allergies. For example, Z4-barley serpin is a possible IgE-binding protein and can evoke a positive skin prick test response (28). A case of beer-induced anaphylaxis was associated to a 38-kDa polypeptides, most likely a fragment of Z-4 barley protein (36). Specific ω- and γ-gliadins are believed the responsible of IgE-mediated atopic eczema/dermatitis syndrome and more severe reactions, such as wheat-dependent exercise-induced anaphylaxis (37). ATIs also are relatively resistant to human gastrointestinal digestion (38). These proteins are believed the environmental factor triggering food-adverse reactions described as “non-celiac gluten sensitivity” (39). Furthermore, ATIs can potentiate the T cell-mediated inflammatory response to gluten in CD (40). Hiemori et al. (41) described an IgE-binding 18 kDa component in beer, which presumably was an ALP. Another group of potential beer allergens identified in all samples is constituted by oleosins, which have hydrophobic domains associated with oil bodies in the caryopsis. Oleosins have already been identified as food allergens in several matrices (42).

**Table 1:** List of CD-toxic proteins and potential IgE-binding food allergens identified in the five beer types using proteomics and peptidomics.

**Protein name**	**Accession** **number**	**Organism**	**Beer_Gluten-free**	**Beer_Hammurabi**	**Beer_ID331**	**All Barley** **Malt Beer**	**Weissbier**
		**Peptide matches (unique)**					
**CD-TOXIC PROTEINS**							
1Bx HMW glutenin subunit	Q1KL95	WHEAT			(HMW)2		
75k γ-secalin	E5KZQ7	WHEAT				(LMW)2	(LMW)1
	E5KZR8		(LMW)5				
	E5KZS2						(LMW)5
	E5KZT8			(HMW)4			(HMW)1
Avenin-3	M8ANS4	TRIUA		(HMW)38	(HMW)36		
Avenin-like a1	Q2A784	WHEAT				(HMW)3	
Avenin-like a2	P0CZ07	WHEAT		(HMW)56			
Avenin-like a4	D2KFH1	WHEAT					(LMW)2-(PSD)2
Avenin-like a6	P0CZ10	WHEAT					(PSD)2
Avenin-like a7	P0CZ11	WHEAT	(HMW)1				
Avenin-like b1	Q2A783	WHEAT					(HMW)33
Avenin-like b2	P0CZ05	WHEAT					(LMW)2-(PSD)4
Avenin-like protein	G9I0U5	WHEAT	(LMW)2				
	V5M0Y3	TRIMO		(HMW)74	(HMW)55		
B1-hordein	P06470	HORVU		(LMW)2			
B3-hordein	I6SJ26	HORVU	(LMW)9				(LMW)9
	I6SW30						(HMW)17
	I6TEV5					(LMW)9	
	I6TMW4				(HMW)14		
	Q4G3S1	HORCH				(HMW)11	
B3-hordein (Fragment)	P06471	HORVU	(LMW)7-(PSD)5	(LMW)4		(HMW)25	
	Q4G3T9	HORCH	(HMW)2				
Barley (*Hordeum vulgare* L.) C-hordein (Fragment)	Q40037	HORVU	(LMW)1				
Barley mRNA for B1-hordein (Fragment)	Q40022	HORVU			(HMW)7		
B-hordein	A0A0K1Z5E8	HORVV					(HMW)5
	I6R4A7						(LMW)3
	Q0PIV6		(PSD)1	(HMW)1	(PSD)5		
	Q2XQF0		(HMW)1				
	Q3LTR1					(LMW)2	(LMW)2
C-hordein	Q40055	HORVU			(HMW)2		(HMW)1
	Q41210		(LMW)3	(LMW)5			(LMW)9
C-hordein (Fragment)	P02864	HORVS				(HMW)3	
	P17991		(LMW)3				(PSD)2
D-hordein	B0L965	HORCH		(LMW)2		(LMW)1	
	I6SW23	HORVU		(LMW)46-(PSD)47		(LMW)50	
	I6SW34	HORVV		(LMW)40		(HMW)70	
	Q40054	HORVU	(LMW)21	(HMW)2	(HMW)53-(LMW)9	(LMW)35	(HMW)33-(LMW)18
D-hordein (Fragment)	Q02056	HORVU	(LMW)3				
	Q40045		(HMW)11				
D-type LMW glutenin subunit (Fragment)	B6ETS0	WHEAT					(HMW)11
Fast ω-gliadin	A0A076G4E3	TRIMO					(HMW)2
	A0A0N9MGE4			(HMW)16			
Gliadin	R4VEK6	WHEAT	(LMW)6		(HMW)17		(LMW)4
Gliadin/avenin-like seed protein	D2KFG9	WHEAT					(HMW)49-(LMW)1
Gliadin/avenin-like seed protein (Fragment)	D2KFH2	WHEAT					(HMW)18-(LMW)2
Glu-B1-1b HMW glutenin subunit	Q42451	WHEAT					(LMW)15
Glutenin, HMW subunit 12	P08488	WHEAT	(HMW)1				
Glutenin, HMW subunit DX5	P10388	WHEAT					(LMW)20
Glutenin, HMW subunit DY10	P10387	WHEAT		(LMW)2			(HMW)65-(LMW)36-(PSD)24
Glutenin, LMW subunit	M7ZXM2	TRIUA		(HMW)34			
Glutenin, LMW subunit 1D1	P10386	WHEAT					(PSD)20
Glutenin, LMW subunit PTDUCD1	P16315	WHEAT					(LMW)2
HMW glutenin	A0A0M3R6K6	TRIMO					(HMW)6
	K4N1X7	TRITD	(LMW)6				
HMW glutenin (Fragment)	Q8H0L1	WHEAT		(HMW)2			
HMW glutenin 1Ax2.1	G0YVZ4	TRIMO	(LMW)35	(HMW)37	(HMW)55		
HMW glutenin protein Dx5 (Fragment)	Q38LF5	WHEAT		(HMW)3			
HMW glutenin subunit	A0A0X9BSF8	WHEAT					(LMW)4
	A0A1D8V7C1				(HMW)13	(HMW)9	
	Q6UKZ5		(LMW)1			(LMW)3	
HMW glutenin subunit (Fragment)	Q7XZB8	WHEAT					(HMW)2
HMW glutenin subunit 1Ay	A0A221HK71	TRIUA					(LMW)2
HMW glutenin subunit 1Ay protein	V9TN80	TRIDC					(LMW)1
HMW glutenin subunit 1Ay/Ta-e3	B5TM09	TRIMO					(HMW)11
HMW glutenin subunit 1By15	W0C8U3	WHEAT	(LMW)5				
HMW glutenin subunit 1By9	Q03871	WHEAT		(HMW)7			
HMW glutenin subunit 1Dy10.1	Q670Q5	WHEAT				(HMW)5	
HMW glutenin subunit Bx17	Q18MZ6	WHEAT		(LMW)2			(LMW)7
HMW glutenin subunit x	Q94IJ7	WHEAT	(HMW)2				
HMW glutenin subunit y	Q94IJ8	WHEAT				(LMW)3	
HMW glutenin x-type subunit Bx7	A0A1G4P219	WHEAT	(LMW)2				
HMW glutenin subunit 1Dx2.2	Q599I0	WHEAT					(LMW)15
HMWglutenin subunit Bx17	Q18MZ6	WHEAT		(HMW)4			(HMW)63
HMWt glutenin y-type (Fragment)	B8XU62	TRIMO	(LMW)5				
Hor1-17 C-hordein	Q40053	HORVU	(LMW)3			(LMW)3	
HRR25-like protein	A0A0L8R9P4	SACEU	(LMW)1				
LMW glutenin subunit D3-1	D3U326	WHEAT					(HMW)25
LMW glutenin subunit group 2 type I	Q8W3X5	WHEAT	(LMW)2				
LMW glutenin subunit LMW-H6-5-2	F6M7E1	WHEAT	(LMW)8				
LMW glutenin	B2Y2Q7	WHEAT					(LMW)32
	C3VN79				(HMW)7		
	Q5MFK8						(HMW)8
	Q5MFQ1		(LMW)2				
	Q7Y074			(HMW)3			(HMW)3
LMW glutenin (Fragment)	A7X9Y0	TRITU					(LMW)2
	Q5MFN4	WHEAT					(HMW)3
LMW glutenin GLU-B3	A7XDG0	TRITU					(HMW)3
LMW glutenin pGL13.1	Q6QGV9	WHEAT	(LMW)2				
LMW glutenin storage protein	P93793	WHEAT			(HMW)2		(HMW)1
	P93794				(PSD)2		
LMW glutenin storage protein (Fragment)	Q7DM83	WHEAT		(HMW)3			
LMW glutenin subunit	A0A0A0QXD3	TRIUA	(LMW)19				
	A4KZ73						(LMW)1
	F8SGM3						(HMW)17
	R4JB53			(HMW)6			
	R4JBL2				(HMW)26		
LMW glutenin subunit (Fragment)	A0A089VKL2	TRIUA				(HMW)1	(LMW)3
	Q9XGF0	TRITD		(HMW)2			
	R4JB40	WHEAT					(HMW)66
	R4JBE8		(LMW)59				
	R4JBK6				(HMW)7		
	R4JFB5						(LMW)7
LMW glutenin subunit Glu-A3 (Fragment)	Q19MN2	WHEAT			(HMW)10		
	X2J8E3		(LMW)16				(LMW)9
LMW glutenin subunit Glu-B3 (Fragment)	X2JAG4	TRITU					(HMW)7
	X2JC81	WHEAT					(LMW)1
LMW glutenin subunit Glu-D3	R9YTM7	WHEAT	(LMW)2-(PSD)2				(LMW)7
LMW glutenin subunit group 3 type II (Fragment)	Q8W3W7	WHEAT		(HMW)3			(HMW)2
	Q8W3X4				(HMW)4		
LMW glutenin subunit group 4 type II	Q8W3W4	WHEAT			(HMW)2		
LMW glutenin subunit group 6 type IV	Q8W3V6	WHEAT		(HMW)14			
LMW glutenin subunit LMW-B8	Q2PQJ8	TRIMO	(LMW)16	(HMW)2			(HMW)3
LMW glutenin subunit LMW-Di31	Q2PQJ7	TRIDC	(LMW)6		(HMW)4		
LMW glutenin subunit LMW-H6-5-2	F6M7E1	WHEAT					(LMW)9
LMW glutenin subunit Y22	Q38L52	TRIDC		(HMW)2			
LMW glutinin subunit	F8SGM9	WHEAT					(LMW)27
LMW protein (Fragment)	C5IFV2	WHEAT			(HMW)4		
LMW-GS	Q6SPZ1	WHEAT				(HMW)3	
	R9XT30						(LMW)7
LMW-m glutenin subunit	D0EVN9	WHEAT					(LMW)8
LMW-m glutenin subunit 5 (Fragment)	V9P6C5	WHEAT					(HMW)2
LMW-m glutenin subunit 7 (Fragment)	V9P7D1	WHEAT					(LMW)4
LMW-m1 glutenin subunit	A2IBJ9	TRIDC	(LMW)2				
Low-molecular-weightLMWsubunit Glu-A3 (Fragment)	X2J8E3	WHEAT					(HMW)5
Pseudo α/β-gliadin	A0A0K2QJB3	WHEAT					(LMW)8
	A0A0K2QJX0			(HMW)35			
Putative LMW glutenin subunit	Q571Q5	WHEAT					(LMW)7
Putative LMW glutenin subunit (Fragment)	A0A0M4FLL7	TRIMO				(HMW)1	
Putative γ-gliadin	Q571R3	WHEAT				(HMW)22	(HMW)10
Putative ω-gliadin (Fragment)	Q571R2	WHEAT	(LMW)1				
Putative ω-secalin	C1KDG3	WHEAT					(LMW)6
	C1KFY6		(LMW)2				
S-type LMW glutenin L4-55 (Fragment)	Q6J160	WHEAT					(LMW)1
Truncated high molecular weight glutenin 1Ay8.3	A0A168M895	TRIMO		(HMW)18	(HMW)20		
Truncated HMW glutenin 1Ay8.1	G0YVZ5	TRIMO	(LMW)12				
Type I LMW-glutenin LMW-i1	A5JJ52	TRIMO			(HMW)62		
X-type HMW glutenin	Q0Q5D2	WHEAT					(HMW)30
Y-type HMW glutenin subunit 1By	M1PIT5	TRIDC					(LMW)4
α/β-gliadin	A0A0K2QJ85	WHEAT			(PSD)1		(LMW)7
	A0A0K2QJC3			(HMW)5	(HMW)9		
	A0A0K2QJD3		(LMW)17				(LMW)11
	A0A0K2QJF4		(LMW)7		(HMW)4		(HMW)12
	I0IT52				(HMW)47		
	P02863					(HMW)1	
α/β-gliadin (Fragment)	D2T2K3	WHEAT		(HMW)16			(HMW)25
α/β-gliadin A-I	P04721	WHEAT		(LMW)2			
α/β-gliadin A-II	P04722	WHEAT	(HMW)2				
α/β-gliadin MM1	M7ZUN8	TRIUA		(LMW)1			
	P18573	WHEAT					(HMW)97-(LMW)58-(PSD)54
α/β-type gliadin	Q41632	TRIUA					(HMW)4-(LMW)1
α-gliadin	A0A023WGC3	TRIMO		(HMW)23			
	D2X6C9	TRITD					(LMW)19
	Q306G0	WHEAT			(HMW)54-(PSD)6		
	R9XUP7						(HMW)57
	X2KVH9		(LMW)37				
α-gliadin (Fragment)	A0A0E3UQU0	WHEAT					(LMW)15
	A0A0E3URD4	TRIMO					(HMW)6
	A0A0E3Z7F3	WHEAT	(LMW)4				
	E7DRL1	TRIMO		(HMW)4	(HMW)2		
	Q41533	WHEAT			(HMW)19		
α-gliadin Gli-1	B5U2V9	TRITI					(HMW)10
α-gliadin Gli2-CN16-12	Q306F9	WHEAT					(HMW)6
α-gliadin protein	A0A0E3X5J7	TRIUA		(HMW)1			(HMW)1
	A0A0E3X5K4	TRIUA	(LMW)2				(LMW)4
	M4WYG5	TRIMO					(HMW)21
	Q2V5Z7	TRITD	(LMW)4				
	X2KWL1	WHEAT			(HMW)13		
α-gliadin storage protein	Q41529	WHEAT		(HMW)4			
α-gliadin storage protein (Fragment)	Q2QL58	TRIMO				(HMW)1	
α-type gliadin (Fragment)	Q3YFI0	TRIDC					(LMW)1
γ-2-gliadin P25-27	Q7M1M5	TRITU		(HMW)3			
γ-gliadin	B5ANT0	WHEAT	(LMW)18				
	B6DQB8			(LMW)2		(LMW)2	
	B6UKM9						(LMW)21
	B6UKS0	TRIUA		(HMW)13	(HMW)23		
	B8XU47	TRIMO			(HMW)74		
	R9XV71	WHEAT					(HMW)72
	U5UA54						(LMW)21
γ-gliadin (Fragment)	B6DQB1	WHEAT					(LMW)10
	B8XU49	TRIMO	(LMW)2		(HMW)32		
	B8XU49		(LMW)5				
	Q1W676	WHEAT				(HMW)1	
γ-gliadin 9	M9TLK0	WHEAT		(HMW)11	(HMW)3		
γ-gliadin B	P06659	WHEAT					(PSD)25
γ-hordein-1	P17990	HORVU	(HMW)6-(PSD)1		(HMW)9		
γ-hordein-3	P80198	HORVU	(HMW)11-(LMW)3	(HMW)1-(LMW)7-(PSD)6	(HMW)28	(HMW)50-(LMW)6	(HMW)26-(LMW)2-(PSD)2
ω-5 gliadin	Q402I5	WHEAT					(LMW)10
ω-gliadin	A0A0B5JD20	WHEAT		(HMW)10			
	D2KKB1	TRITU		(LMW)2			
	Q0GK30	TRITI	(LMW)40				
	U5U6L8	WHEAT					(LMW)4
ω-gliadin (Fragment)	C0KEI1	WHEAT			(HMW)2	(HMW)2	
	C0KEI2						(LMW)5
	D6QY47	TRIMO	(LMW)12	(HMW)2	(HMW)4		(LMW)3
ω-gliadin protein	A0A0E3SZN6	TRIUA	(LMW)5				
ω-secalin	A0A159KI54	WHEAT				(HMW)1	(HMW)9
	C4NFQ1						(LMW)17
**IGE FOOD ALLERGENS**							
7 kDa lipid transfer protein	O81135	HORVU				(HMW)1	
Barley trypsin inhibitor CMc (Fragment)	E7BB45	HORVV				(HMW)4	
Bowman-Birk type trypsin inhibitor	M0Y075	HORVV				(HMW)1	
	P81713	WHEAT					(HMW)2
Chymotrypsin inhibitor WCI	P83207	WHEAT					(HMW)4
CMd subunit of tetrameric α-amylase inhibitor	O23982	HORVU	(PSD)1				
Dimeric α-amylase inhibitor	C3VW72	TRIDC			(HMW)2		
	C3VWA4			(HMW)6			
	C3VWG2						(HMW)24
Dimeric α-amylase inhibitor (Fragment)	A4GFP2	TRIDC			(HMW)8		
	A4GFQ9						(HMW)2
	M1PST7	TRIUA		(HMW)1			
Endogenous α-amylase/subtilisin inhibitor	P16347	WHEAT		(HMW)3	(HMW)3		(HMW)10-(LMW)11
	P16347						(PSD)13
Monomeric α-amylase inhibitor	C4P622	TRIDC					(HMW)18
Non-specific lipid-transfer protein	A0A1D5RXY7	WHEAT	(HMW)2				(HMW)1
	A0A1D5YFR5			(HMW)12	(HMW)18		
	Q5UNP2	HORVV			(HMW)3	(HMW)12	(HMW)6
	T1MH09	TRIUA	(LMW)1				
Non-specific lipid-transfer protein (Fragment)	P24296	WHEAT		(HMW)3	(HMW)3		(LMW)3-(PSD)2
Non-specific lipid-transfer protein 1	P07597	HORVU	(HMW)31-(LMW)3-(PSD)4	(HMW)12-(LMW)4-(PSD)3	(HMW)47-(LMW)4	(HMW)57-(LMW)4	(HMW)34-(LMW)3
Nonspecific lipid-transfer protein 2, putative, expressed	Q337E3	ORYSJ		(HMW)2			(HMW)2
Non-specific lipid-transfer protein 2G	M7YYL5	TRIUA					(HMW)3
	P82900	WHEAT	(HMW)7		(HMW)15		
Non-specific lipid-transfer protein 2P	P82901	WHEAT		(HMW)21		(HMW)6	(HMW)14
Non-specific lipid-transfer protein 4.3	Q42842	HORVU					(HMW)2
Oleosin	F2E8X4	HORVV	(PSD)1		(HMW)1	(HMW)4	
	I3NM41	WHEAT					(HMW)3-(LMW)5
	Q43769	HORVU	(LMW)7	(LMW)5		(HMW)3	
	Q43770		(HMW)1	(LMW)2-(PSD)2	(HMW)2	(HMW)1	
Oleosin (Fragment)	Q43474	HORVU		(LMW)1	(HMW)2		
Probable non-specific lipid-transfer protein	P20145	HORVU	(HMW)22	(HMW)8	(HMW)26	(HMW)27	(HMW)14
Probable secreted beta-glucosidase SIM1	P40472	YEAST				(HMW)4	
Serpin 3	C0LF32	WHEAT					(HMW)46
Serpin-Z1B	P93693	WHEAT	(HMW)1			(HMW)1	(LMW)5
Serpin-Z1C	M7ZQF1	TRIUA			(HMW)27		
Serpin-Z2B	P93692	WHEAT					(LMW)2
Serpin-Z4	M0UEE6	HORVV	(HMW)22		(HMW)73	(HMW)65-(LMW)3	(HMW)44
	P06293	HORVU	(LMW)2-(PSD)2	(HMW)30-(LMW)3-(PSD)3			(LMW)2
Serpin-Z7	M8A993	TRIUA	(HMW)1			(HMW)4	
	Q43492	HORVU		(LMW)1-(PSD)1			
Starch synthase, chloroplastic/amyloplastic	Q8H1Y7	HORVV			(HMW)2		
Subtilisin-chymotrypsin inhibitor CI-1A	P16062	HORVU			(HMW)3	(HMW)4	(HMW)3
Subtilisin-chymotrypsin inhibitor CI-1B	P16063	HORVU	(PSD)1				
Subtilisin-chymotrypsin inhibitor-2A	P01053	HORVU					(HMW)1
Trypsin inhibitor	C5J3R4	TRIMO		(HMW)19	(HMW)9		(HMW)1
Trypsin inhibitor CMc	P34951	HORVU	(LMW)2				
Trypsin inhibitor CMe	M0UY52	HORVV					(HMW)13
Trypsin inhibitor Cme	P01086	HORVU	(HMW)5	(HMW)3	(HMW)15	(HMW)19	
Z-like serpin (Fragment)	Q9S8N4	WHEAT			(HMW)5		(HMW)6
α/β-amylase	Q84T20	HORVV				(HMW)32	
α-amylase inhibitor (Fragment)	Q7M219	TRITD					(HMW)2
α-amylase inhibitor 0.19	P01085	WHEAT					(HMW)40
α-amylase inhibitor 0.28	P01083	WHEAT		(HMW)2			(LMW)3-(PSD)5
α-amylase inhibitor 0.53	P01084	WHEAT					(LMW)1
α-amylase inhibitor BDAI-1	P13691	HORVU	(HMW)11	(HMW)13	(HMW)30	(HMW)28	(HMW)20
α-amylase inhibitor BMAI-1 (Fragment)	P16968	HORVU	(HMW)2		(HMW)2	(HMW)10	(HMW)4
α-amylase type A isozyme	P00693	HORVU					(HMW)1
α-amylase type B isozyme	P04063	HORVU	(PSD)1	(LMW)1		(HMW)4	
α-amylase/subtilisin inhibitor	P07596	HORVU	(HMW)1-(LMW)2-(PSD)1	(LMW)8-(PSD)8	(HMW)4-(LMW)1	(HMW)6-(LMW)9	(HMW)2-(LMW)2
α-amylase/trypsin inhibitor	P16969	HORVU					(HMW)1
α-amylase/trypsin inhibitor CM1	P16850	WHEAT		(HMW)2			
α-amylase/trypsin inhibitor CM16	P16159	WHEAT	(HMW)4				(HMW)18
α-amylase/trypsin inhibitor CM2	P16851	WHEAT					(HMW)30
α-amylase/trypsin inhibitor CM3	P17314	WHEAT		(HMW)2			
α-amylase/trypsin inhibitor Cma	P28041	HORVU	(HMW)5	(HMW)4	(HMW)18	(HMW)27	
α-amylase/trypsin inhibitor CMb	P32936	HORVU		(HMW)7	(HMW)19	(HMW)18	
α-amylase/trypsin inhibitor CMd	M0Y227	HORVV					(HMW)19
	P11643	HORVU	(HMW)4		(HMW)14	(HMW)33	
β-amylase	A0A1D5YFA7	WHEAT		(HMW)4			
	P16098	HORVU	(HMW)4-(LMW)6-(PSD)1	(HMW)2	(HMW)14	(LMW)10	
	P82993	HORVS	(LMW)4	(LMW)6-(PSD)6		(LMW)2	(LMW)3-(PSD)3
	W5EKI0	WHEAT	(LMW)3	(LMW)1	(PSD)1		(HMW)8-(LMW)4
β-amylase (Fragment)	Q7X9M2	WHEAT			(HMW)2		
	Q9SB23	HORVU					(HMW)1

HMW and LMW indicate if proteins were detected in the high- or low-molecular weight fractions, respectively, along with the number of unique peptides identified. PSD indicates the peptides belonging to a protein entry detected “post simulated digestion.”

The occurrence of gluten-derived proteins/peptides also including CD-toxic epitopes has been largely demonstrated and confirmed by this study in the five types of beer, also including the one labeled as “gluten-free.” In agreement with previous findings, peptidomic analysis confirmed that PEP enzymes are not able to completely hydrolyze all the immunopathogenic peptides of gluten (43). The accurate quantification of gluten epitopes remains a main analytical concern, because the current immunochemical methods used to assess gluten in food have limited reliability for beer and other extensively processed products (13, 15, 33, 44). The unavailability of trustful determinations for the most consumed beer types hampers estimating the contribution of beer consumption to the extent of intestinal tissue damages.

### Simulated Gastroduodenal Digestion

Gastric and intestinal proteases profoundly degrade dietary proteins. While for IgE-mediated allergies sensitizing/eliciting capability of food allergens appears definitely independent from their digestion stability (45), the CD etiopathogenesis implies that determinants, i.e., gluten peptides, survive digestion and translocate the intestinal epithelium.

Bioaccessibility of beer polypeptides has not been investigated so far. Thus, the five beer types were subjected to a simulated GD digestion and the resulting digests analyzed by the G12 ELISA assay and by HPLC-MS/MS.

Gluten amount before and after digestion was determined by the G12 competitive ELISA assay. It has demonstrated that the results obtained with the G12 antibody ELISA assay are comparable to those of the official R5 method (46). However, G12 could be more appropriate to specifically quantify immunotoxic peptides, because it is based on a monoclonal antibody developed against gluten epitopes contained in the α-gliadin immunodominant 33-mer peptide (47).

Gluten estimated by G12 assay was > 80 ppm in Weiss, Hammurabi and ID331, and < 20 ppm in all-barley malt (16 ppm) and gluten-free beer (11 ppm) types. After GD simulated digestion, gluten values were abundantly below the threshold for a food to be considered “gluten-free” (20 ppm) in all the beer samples. The reported values are average of three independent determinations, with relative standard deviation <10% in all cases.

Although gluten should be more reliably quantified with antibody-independent techniques (e.g., MS), the HPLC-MS/MS analysis of beer peptide digests clearly showed a substantial reduction of all protein components with respect to the undigested samples. Interestingly, no beer-derived protein bands were detected by SDS-PAGE analysis after gastrointestinal digestion (data not shown), indicating that large polypeptides were hydrolyzed. Peptides surviving GD digestion in all beer types are summarized in Table S4. Peptides are also grouped according to their origin from CD-toxic proteins or IgE-binding food allergens (Table S4). Most of the protein allergens surviving malting and released into wort can be denatured during the various brewing steps. This might explain the high susceptibility of metabolic proteins to GD simulated degradation. In contrast, several gluten-derived peptides were detected in digests of Weissbier, Hammurabi and ID331 beers, though their number was significantly reduced compared to the undigested samples. Digests of all-barley malt and gluten-free beers did not contain detectable gluten-like epitopes, though a few fragments of hordein occurred. The partial proteolytic susceptibility of gluten-like (poly)peptides could be the effect of a relatively high proteases-to-substrate ratio during simulated digestion, rather than the consequence of thermal-induced conformational transitions, since these peptides reasonably lack defined and stable tertiary structure. In all cases the TIC chromatograms of peptides from digested beer showed a drastic decrease of intensity compared to the undigested counterparts, in agreement with previous observations (48).

### Stimulatory Properties of Beer Polypeptides on Celiac T Cells

**Figure 2:**
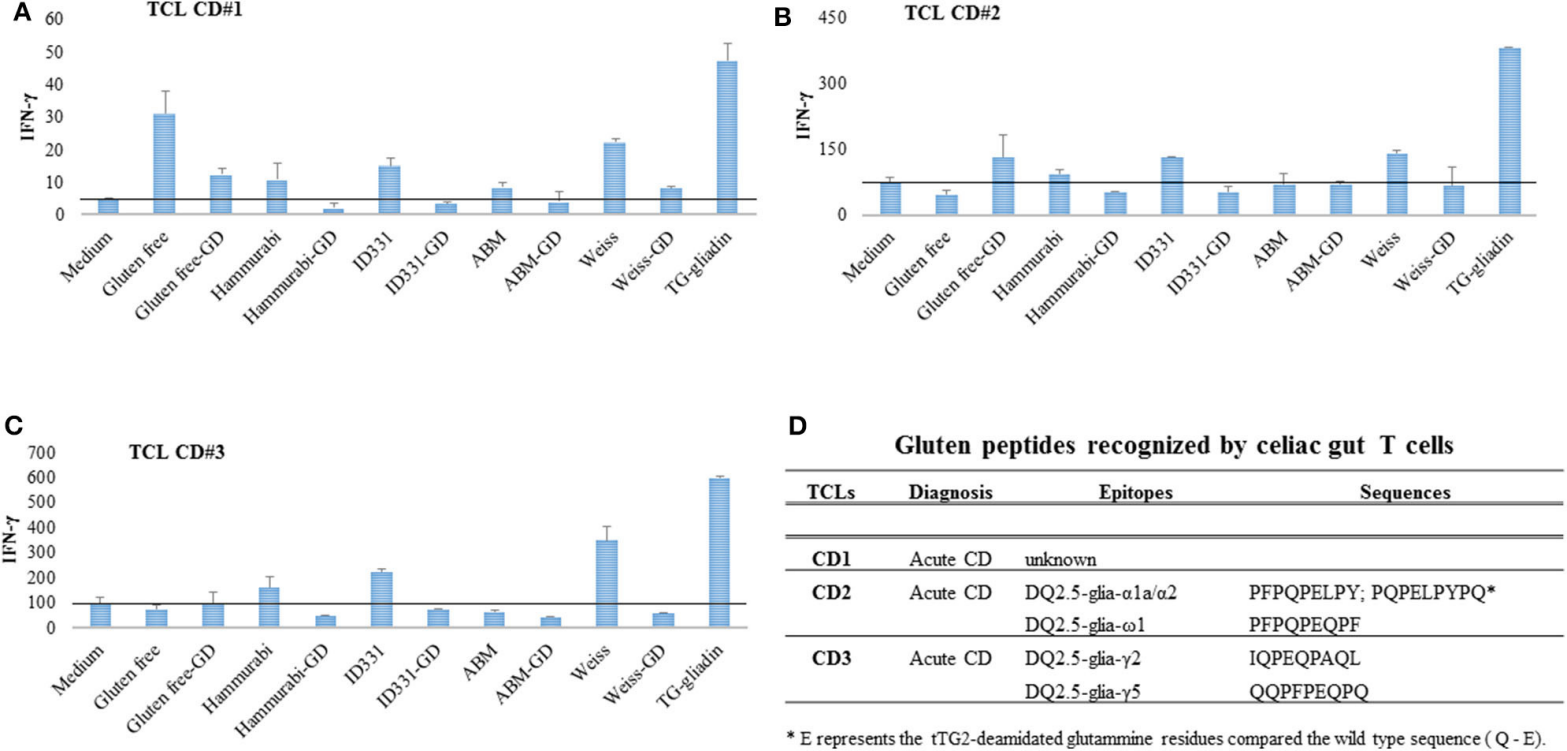
The immunogenic potential of beer samples was assessed on gliadin-reactive T cell lines established from gut mucosa of three CD patients. Beer peptides, deamidated with tTG, were assessed for immunogenicity before and after simulated gastrointestinal digestion. **(A–C)** INF-γ response of intestinal T cell lines established from three different patients with acute CD. The horizontal line indicates for each CD the average level of INF-γ activation by medium. **(D)** Clinical information and repertoire of gluten peptides recognized by each celiac T cell line used to assess beer bioactivity. ABM, all-barley malt beer; GD, gastroduodenal digests.

The capability of selected beers to elicit adaptive immune response was evaluated on T cells (TCL) obtained from intestinal biopsies of CD patients (Figures 2A–C) and reactive to the most immunogenic gliadin peptides (Figure 2D). Beer peptides extracted from untreated and GD hydrolyzed beer samples were used to stimulate *in vitro* TCL from three CD patients. Peptides were deamidated by tTG prior to be used as antigens in IFN-γ-production read-out assay. As showed in Figure 2, all the TCL analyzed were highly reactive to PC-gliadin, used as internal comparative control. Overall, the beer samples were weakly immunogenic, as they induced low IFN-γ responses, comparably to medium. In particular, the Weissbier was the most active on the three CD TCL, which is consistent with the relatively high abundance of specific immunoreactive epitopes. Because of the presence of immunotoxic peptides, the gluten-free beer induced a substantial IFN-γ response, especially in CD#1, in agreement with a previous observation with gluten-reduced beer (49). Nevertheless, in the case of gluten-free beer the levels of INF-γ was drastically reduced by the simulated GD digestion in one (CD#1), while it increased in CD#2 and CD#3, although these increments were not statistically significant. The *p*-values for two tail paired Student's *t*-test are reported in Table S5. The immune response to gluten epitopes is variable, depending on the individual immune asset (Figure 2D). In principle, GD digestion might increase the immunotoxicity releasing epitopes formerly encrypted in longer polypeptides. Furthermore, GD digestion may affect the balance of beer epitopes, destroying some sensitive sequences while preserving the digestion-resistant ones. Dedicate studies with a higher number of CD subjects and accurate epitope quantification should contribute to clarify these aspects.

Like Weissbier, the beer samples crafted with Hammurabi and ID331 einkorn lines were more immunogenic than all-barley malt beer, though in all these cases the bioactivity was sensitive to GD hydrolysis. Overall, our findings demonstrated that beer samples activate, although to a very low extent, an immune response on celiac TCL, which was markedly reduced upon GD digestion, consistently with peptidomic data about the substantial degradation of gluten immunogenic sequences.

## Conclusions

Beer contains a great variety of allergenic and CD-eliciting proteins and/or derived proteolytic fragments as assessed by proteomics and peptidomics. Notably, beer types labeled as “gluten-free” do contain gluten-derived epitopes as well, at amounts that remain to be accurately determined. However, both allergenic and CD-toxic polypeptides of beer are heavily degraded by gastrointestinal proteases. Gluten peptides appeared particularly degraded in beer samples brewed with *T. monococcum* malt.

In general, susceptibility to GD proteolysis reduces the CD-eliciting potential of gluten toxic epitopes, and might decrease the allergenicity in relation to adverse reactions with immunopathological mechanisms other than CD. This finding is consistent with the low prevalence of severe adverse reactions to beer, despite the multiplicity of allergens contained and the elevated average consume of this beverage worldwide.

It needs to be emphasized that beer brewed with *Hordeum* or *Triticum* spp. malt is not safe for celiacs, and it should be precautionarily excluded from their diet. On the other hand, gluten content of beer can vary within very ample ranges, according to the raw material and to the brewing process. Although univocal data obtained with non-immunochemical methods are still missing, many commercial (conventional barley malt) beer samples are believed to contain gluten-like epitopes at level < 20 ppm (50, 51). Therefore, considering the relatively low amount of total proteinaceous material in beer and the severe degradation that gluten peptides undergo during gastrointestinal digestion, a moderate uptake of beer with *Hordeum* and/or *T. monococcum* malt could supply relatively low levels of gluten. The findings of this study highlight the urgent demand of reliable methods for accurate quantification of gluten in processed foods and beverages. The suitability of commercial beer crafted with barley and labeled as “gluten-free” should be carefully evaluated. In general, opportune *in vivo* trials should be also designed to relate the overall intake of beer gluten-like peptides with the eventual entity of the damage induced on intestinal mucosa of CD individuals.

## Data Availability Statement

All datasets for this study are included in the article/Supplementary Material.

## Author Contributions

VS and LD prepared samples for analysis. LD determined gluten amount with immunochemical methods. GM and GP performed the proteomic experiments. VS elaborated the manuscript data. BM selected the cereal varieties and brewed the einkorn beer samples. SP and CG carried out T cell assays. GP conceived the research and wrote the paper with the contribution of all the coauthors. All authors contributed to the article and approved the submitted version.

## Conflict of Interest

The authors declare that the research was conducted in the absence of any commercial or financial relationships that could be construed as a potential conflict of interest.

## References

[1] Leiper KA Miedl M Colloidal stability of beer. In: Bamforth CW Russell I Stewart G editors. Handbook of Alcoholic Beverages Series, Beer: A Quality Perspective. San Diego, CA: Elsevier Ltd (2009). p. 111–61. 10.1016/B978-0-12-669201-3.00004-X

[2] Picariello G Mamone G Cutignano A Fontana A Zurlo L Addeo F. Proteomics, peptidomics, and immunogenic potential of wheat beer (Weissbier). J Agric Food Chem. (2015) 63:3579–86. 10.1021/acs.jafc.5b00631 25793656

[3] Picariello G Bonomi F Iametti S Rasmussen P Pepe C Lilla S Proteomic and peptidomic characterization of beer: immunological and technological implications. Food Chem. (2011) 124:1718–26. 10.1016/j.foodchem.2010.07.111

[4] Ellis HJ Freedman AR Ciclitira PJ. Detection and estimation of the barley prolamin content of beer and malt to assess their suitability for patients with coeliac disease. Clin Chim Acta. (1990) 189:123–30. 10.1016/0009-8981(90)90082-4 2204500

[5] Perrocheau L Rogniaux H Boivin P Marion D. Probing heat-stable water-soluble proteins from barley to malt and beer. Proteomics. (2005) 5:2849–58. 10.1002/pmic.200401153 15986330

[6] Iimure T Nankaku N Hirota N Tiansu Z Hoki T Kihara M Construction of a novel beer proteome map and its use in beer quality control. Food Chem. (2010) 118:566–74. 10.1016/j.foodchem.2009.05.022

[7] Konečná H Müller L Dosoudilová H Potěšil D Buršíková J Sedo O. Exploration of beer proteome using OFFGEL prefractionation in combination with two-dimensional gel electrophoresis with narrow pH range gradients. J Agric Food Chem. (2012) 60:2418–26. 10.1021/jf204475e 22353030

[8] Picariello G Mamone G Nitride C Addeo F Camarca A Vocca I. Ferranti P. Shotgun proteome analysis of beer and the immunogenic potential of beer polypeptides. J. Proteomics. (2012) 75:5872–82. 10.1016/j.jprot.2012.07.038 22868252

[9] Colgrave ML Goswami H Howitt CA Tanner GJ What is in a beer? Proteomic characterization and relative quantification of hordein (gluten) in beer. J Proteome Res. (2012) 11:386–96. 10.1021/pr2008434 21999962

[10] Grochalová M Konečná H Stejskal K Potěšil D Fridrichová D Srbová E. Deep coverage of the beer proteome. J Proteomics. (2017) 162:119–24. 10.1016/j.jprot.2017.05.001 28478308

[11] Sun Z Yu X Zhang Y Xu J Li X. Construction of a comprehensive beer proteome map using sequential filter-aided sample preparation coupled with liquid chromatography tandem mass spectrometry. J Separation Sci. (2019) 42:2835–41. 10.1002/jssc.201900074 31218791

[12] Picariello G Mamone G Nitride C Ferranti P Proteomic analysis of beer. In: Colgrave ML editor. Proteomics in Food Science: From Farm to Fork. London, UK: Academic Press-Elsevier (2017) p. 383–403. 10.1016/B978-0-12-804007-2.00023-0

[13] Fiedler KL Panda R Croley TR. Analysis of gluten in a wheat-gluten-incurred Sorghum beer brewed in the presence of proline endopeptidase by LC/MS/MS. Anal Chem. (2018) 90:2111–8. 10.1021/acs.analchem.7b04371 29328628

[14] Panda R Garber EAE. Detection and quantitation of gluten in fermented-hydrolyzed foods by antibody-based methods: challenges, progress, and a potential path forward. Front Nutr. (2019) 6:97. 10.3389/fnut.2019.00097 31316993 PMC6611335

[15] Tanner GJ Colgrave ML Blundell MJ Goswami HP Howitt CA. Measuring hordein (gluten) in beer–a comparison of ELISA and mass spectrometry. PLoS ONE. (2013) 8:e56452. 10.1371/journal.pone.0056452 23509606 PMC3585340

[16] Sollid LM Qiao S-W Anderson RP Gianfrani C Koning F. Nomenclature and listing of celiac disease relevant gluten T-cell epitopes restricted by HLA-DQ molecules. Immunogenetics. (2012) 64:455–60. 10.1007/s00251-012-0599-z 22322673 PMC3349865

[17] Sollid LM Tye-Din JA Qiao SW Anderson RP Gianfrani C Koning F. Update 2020: nomenclature and listing of celiac disease-relevant gluten epitopes recognized by CD4(+) T cells. Immunogenetics. (2019) 72:85–8. 10.1007/s00251-019-01141-w 31735991

[18] Petersen J Montserrat V Mujico JR Loh KL Beringer DX van Lummel M. T-cell receptor recognition of HLA-DQ2-gliadin complexes associated with celiac disease. Nat Struct Mol Biol. (2014) 21:480–8. 10.1038/nsmb.2817 24777060

[19] Scherf KA Wieser H Koehler P. Novel approaches for enzymatic gluten degradation to create high-quality gluten-free products. Food Res Int. (2018) 110:62–72. 10.1016/j.foodres.2016.11.021 30029707

[20] Hager AS Taylor JP Waters DM Arendt EK Gluten-free beer – a review. Trends Food Sci Technol. (2014) 36:44–54. 10.1016/j.tifs.2014.01.001

[21] Gianfrani C Camarca A Mazzarella G Di Stasio L Giardullo N Ferranti P. Extensive in vitro gastrointestinal digestion markedly reduces the immune-toxicity of *Triticum monococcum* wheat: implication for celiac disease. Mol Nutr Food Res. (2015) 59:1844–54. 10.1002/mnfr.201500126 26016626

[22] Gianfrani C Maglio M Rotondi Aufiero V Camarca A Vocca I Iaquinto G. Immunogenicity of monococcum wheat in celiac patients. Am J Clin Nutr. (2012) 96:1339–45. 10.3945/ajcn.112.040485 23134879

[23] Mamone G Addeo F Chianese L Di Luccia A de Martino A Nappo A. Characterization of wheat gliadin proteins by combined two-dimensional gel electrophoresis and tandem mass spectrometry. Proteomics. (2005) 5:2859–65. 10.1002/pmic.200401168 15952231

[24] Minekus M Alminger M Alvito P Ballance S Bohn T Bourlieu C. A standardised static in vitro digestion method suitable for food - an international consensus. Food Funct. (2014) 5:1113–24. 10.1039/C3FO60702J 24803111

[25] Brodkorb A Egger L Alminger M Alvito P Assunção R Ballance S. INFOGEST static in vitro simulation of gastrointestinal food digestion. Nat Protoc. (2019) 14:991–1014. 10.1038/s41596-018-0119-1 30886367

[26] Camarca A Anderson RP Mamone G Fierro O Facchiano A Costantini S. Intestinal T cell responses to gluten peptides are largely heterogeneous: implications for a peptide-based therapy in celiac disease. J Immunol. (2009) 182:4158–66. 10.4049/jimmunol.0803181 19299713 PMC3306175

[27] Camarca A Auricchio R Picascia S Fierro O Maglio M Miele E. Gliadin-reactive T cells in Italian children from prevent CD cohort at high risk of celiac disease. Pediatr Allergy Immunol. (2017) 28:362–9. 10.1111/pai.12720 28339124

[28] García-Casado G Crespo JF Rodríguez J Salcedo G. Isolation and characterization of barley lipid transfer protein and protein Z as beer allergens. J Allergy Clin Immunol. (2001) 108:647–9. 10.1067/mai.2001.118793 11590395

[29] Weber D Cléroux C Godefroy SB. Emerging analytical methods to determine gluten markers in processed foods–method development in support of standard setting. Anal Bioanal Chem. (2009) 395:111–7. 10.1007/s00216-009-2943-1 19636545 PMC2724643

[30] Iimure T Kihara M Sato K Ogushi K. Purification of barley dimeric α-amylase inhibitor-1 (BDAI-1) and avenin-like protein-a (ALP) from beer and their impact on beer foam stability. Food Chem. (2015) 172:257–64. 10.1016/j.foodchem.2014.09.012 25442552

[31] Bhattacharya S Dhar S Banerjee A Ray S. Structural, functional, and evolutionary analysis of late embryogenesis abundant proteins (LEA) in *Triticum aestivum*: a detailed molecular level biochemistry using *in silico* approach. Comput Biol Chem. (2019) 82:9–24. 10.1016/j.compbiolchem.2019.06.005 31247397

[32] Colgrave ML Byrne K Howitt CA. Liquid chromatography-mass spectrometry analysis reveals hydrolyzed gluten in beers crafted to remove gluten. J Agric Food Chem. (2017) 65:9715–25. 10.1021/acs.jafc.7b03742 29047268

[33] Colgrave ML Goswami H Blundell M Howitt CA Tanner GJ. Using mass spectrometry to detect hydrolysed gluten in beer that is responsible for false negatives by ELISA. J Chromatogr A. (2014) 1370:105–14. 10.1016/j.chroma.2014.10.033 25454134

[34] Fraser JS Engel W Ellis HJ Moodie SJ Pollock EL Wieser H. Coeliac disease: in vivo toxicity of the putative immunodominant epitope. Gut. (2003) 52:1698–702. 10.1136/gut.52.12.1698 14633945 PMC1773874

[35] Finkina EI Melnikova DN Bogdanov IV Ovchinnikova TV. Lipid transfer proteins as components of the plant innate immune system: structure, functions, and applications. Acta Naturae. (2016) 8:47–61. 10.32607/20758251-2016-8-2-47-61 27437139 PMC4947988

[36] Figueredo E Quirce S del Amo A Cuesta J Arrieta I Lahoz C. Beer-induced anaphylaxis: identification of allergens. Allergy. (1999) 54:630–4. 10.1034/j.1398-9995.1999.00827.x 10435480

[37] Battais F Mothes T Moneret-Vautrin DA Pineau F Kanny G Popineau Y. Identification of IgE-binding epitopes on gliadins for patients with food allergy to wheat. Allergy. (2005) 60:815–21. 10.1111/j.1398-9995.2005.00795.x 15876313

[38] Mamone G Nitride C Picariello G Addeo F Ferranti P Mackie A. Tracking the fate of pasta *(T. durum semolina)* immunogenic proteins by *in vitro* simulated digestion. J Agric Food Chem. (2015) 63:2660–7. 10.1021/jf505461x 25682706

[39] Biesiekierski JR Iven J. Non-coeliac gluten sensitivity: piecing the puzzle together. United European Gastroenterol J. (2015) 3:160–5. 10.1177/2050640615578388 25922675 PMC4406911

[40] Junker Y Zeissig S Kim SJ Barisani D Wieser H Leffler DA. Wheat amylase trypsin inhibitors drive intestinal inflammation via activation of toll-like receptor 4. J Exp Med. (2012) 209:2395–408. 10.1084/jem.20102660 23209313 PMC3526354

[41] Hiemori M Eguchi Y Kimoto M Yamasita H Takahashi K Takahashi K. Characterization of new 18-kDa IgE-binding proteins in beer. Biosci Biotechnol Biochem. (2008) 72:1095–8. 10.1271/bbb.70584 18391472

[42] Mills EN Johnson P Alexeev Y Breiteneder H. Identification and characterization of food allergens. In: Coutts J editor. Management of Food Allergens. Oxford, UK: Wiley-Blackwell (2009). p. 42–69. 10.1002/9781444309911.ch3

[43] Panda R Zoerb HF Cho CY Jackson LS Garber EA. Detection and quantification of gluten during the brewing and fermentation of beer using antibody-based technologies. J Food Prot. (2015) 78:1167–77. 10.4315/0362-028X.JFP-14-546 26038908

[44] Panda R Fiedler KL Cho CY Cheng R Stutts WL Jackson LS. Effects of a proline endopeptidase on the detection and quantitation of gluten by antibody-based methods during the fermentation of a model Sorghum beer. J Agric Food Chem. (2015) 63:10525–35. 10.1021/acs.jafc.5b04205 26548701

[45] Verhoeckx K Bøgh KL Dupont D Egger L Gadermaier G Larré C. The relevance of a digestibility evaluation in the allergenicity risk assessment of novel proteins. Opinion of a joint initiative of COST action ImpARAS and COST action INFOGEST. Food Chem Toxicol. (2019) 129:405–23. 10.1016/j.fct.2019.04.052 31063834

[46] Hochegger R Mayer W Prochaska M. Comparison of R5 and G12 antibody-based ELISA used for the determination of the gluten content in official food samples. Foods. (2015) 4:654–64. 10.3390/foods4040654 28231228 PMC5224559

[47] Comino I Real A Moreno Mde L Montes R Cebolla A Sousa C. Immunological determination of gliadin 33-mer equivalent peptides in beers as a specific and practical analytical method to assess safety for celiac patients. J Sci Food Agric. (2013) 93:933–43. 10.1002/jsfa.5830 22886585

[48] Juhász A Gell G Békés F Balázs E. The epitopes in wheat proteins for defining toxic units relevant to human health. Funct Integr Genomics. (2012) 12:585–98. 10.1007/s10142-012-0302-3 23179564

[49] Allred LK Lesko K McKiernan D Kupper C Guandalini S. The celiac patient antibody response to conventional and gluten-Removed Beer. J AOAC Int. (2017) 100:485–91. 10.5740/jaoacint.16-0184 28118560

[50] Dostálek P Hochel I Méndez E Hernando A Gabrovská D. Immunochemical determination of gluten in malts and beers. Food Addit Contam. (2006) 23:1074–8. 10.1080/02652030600740637 17071509

[51] Guerdrum LJ Bamforth CW Levels of gliadins in commercial beers. Food Chem. (2011) 129:5–6. 10.1016/j.foodchem.2011.06.021

