# Supplementary material for: Immunogenic Potential of Beer Types Brewed With Hordeum and Triticum spp. Malt Disclosed by Proteomics
Source: Front Nutr. 2020 Jul 9;7:98. doi: 10.3389/fnut.2020.00098 (PMC7363779; doi:10.3389/fnut.2020.00098)
Supplement: Supplementary file 5 [file Table_5.DOCX]

**Table S5**. Two tail paired Student’s t-test relevant to the INF-g response by T-cell lines obtained from CD subjects. Significance level was set at p<0.05.

| **t-test** | **CD#1**  **(*p-*value)** | **CD#2**  **(*p-*value)** | **CD#3**  **(*p-*value)** |
| --- | --- | --- | --- |
| Gluten free *vs* Gluten free-GD | 0.06 | 0.19 | 0.44 |
| Hammurabi *vs* Hammurabi-GD | 0.27 | 0.36 | 0.03 |
| ID331 *vs* ID331-GD | 0.02 | 0.02 | 0.01 |
| Barley malt *vs* Barley malt-GD | 0.09 | 0.42 | 0.42 |
| Weiss *vs* Weiss-GD | 0.001 | 0.06 | 0.05 |
